# Influence of recurrent interactions on texture processing in networks with different visual map organizations

**DOI:** 10.1186/1471-2202-16-S1-P115

**Published:** 2015-12-18

**Authors:** Hanna Kamyshanska, Dmitry Bibichkov, Matthias Kaschube

**Affiliations:** 1 Frankfurt Institute for Advanced Studies and Faculty of Computer Science and Mathematics, Johann Wolfgang Goethe University, Frankfurt am Main, 60438, Germany

## 

The functional architecture of the visual cortex displays marked differences across mammalian species: in stark contrast to primates, in which the preferred stimulus orientation forms an almost smooth map across the cortical surface, in rodents a 'salt-and-pepper' organization has been observed [1]. It is conceivable that the organization of preferred orientation has an impact on the processing of visual input. Recently [2], we found that in a biologically inspired object recognition system with a pure feed forward network architecture, smooth orientation maps outperform the salt-and-pepper organization in a texture recognition task. Here, we extend this work to study the effect of recurrent connections on neuronal response properties and texture recognition, comparing the two types of cortical architectures.

Our model is a recurrent single-layer rate network. The feed forward connections to each neuron are properly oriented Gabor-filters. Recurrent connections between two neurons depend both on the spatial distance between these neurons and on their orientation selectivities. Inspired by [3], the angle-dependent interaction function is weighted by the product of selectivities of both neurons and enables excitation between cells with similar preferred orientations and inhibition between cells with orthogonal ones. We also study the effects of purely distant-dependent recurrent connectivity of Mexican-hat type, with spatial extent related to local column spacing in case of smooth map layout. We design a network for the salt-and-pepper organization in an analogous way, assuming the same spatial extent of connectivity and its tuning-dependence as observed in mouse visual cortex [4].

Varying the strength and selectivity of recurrent versus feed-forward connections, we first explore the influence of recurrence on the orientation selectivity. For that purpose, we drive the network with oriented gratings to reconstruct the selectivity from the activities. In agreement with [5] we observe sharpening of the orientation tuning of the network by recurrent interactions, such that oriented stimuli can be well discriminated even with weak feed-forward tuning. We further study the role of recurrent connections in processing more complex stimuli. We present visual textures to the model, then feed the responses into a classifier (linear SVM) that learns to predict a class label. This allows us to study how differences in feed-forward and recurrent connections impact texture classification, and to compare the orientation-preference map and salt-and-pepper organization in texture recognition tasks.
